# Introduction and spread of variegated squirrel bornavirus 1 (VSBV-1) between exotic squirrels and spill-over infections to humans in Germany

**DOI:** 10.1080/22221751.2021.1902752

**Published:** 2021-03-30

**Authors:** Daniel Cadar, Valerie Allendorf, Vanessa Schulze, Rainer G. Ulrich, Kore Schlottau, Arnt Ebinger, Bernd Hoffmann, Donata Hoffmann, Dennis Rubbenstroth, Gabriele Ismer, Chris Kibbey, Anna Marthaler, Jürgen Rissland, Frank Leypoldt, Martin Stangel, Jonas Schmidt-Chanasit, Franz J. Conraths, Martin Beer, Timo Homeier-Bachmann, Dennis Tappe

**Affiliations:** a Bernhard Nocht Institute for Tropical Medicine, Hamburg, Germany; b Friedrich-Loeffler-Institut, Federal Research Institute for Animal Health, Institute of Epidemiology, Greifswald-Insel Riems, Germany; c Friedrich-Loeffler-Institut, Federal Research Institute for Animal Health, Institute of Novel and Emerging Infectious Diseases, Greifswald-Insel Riems, Germany; d Friedrich-Loeffler-Institut, Federal Research Institute for Animal Health, Institute of Diagnostic Virology, Greifswald-Insel Riems, Germany; e Animal Park, Schleswig-Holstein, Germany; f Cotswold Wildlife Park and Gardens, Burford, Oxfordshire, UK; g Institute for Virology, Universität des Saarlandes, Homburg/Saar, Germany; h Division of Neuroimmunology, Institute of Clinical Chemistry, Universitätsklinikum Schleswig-Holstein, Kiel, Germany; i Department of Clinical Neuroimmunology and Neurochemistry, and Department of Neurology, Medizinische Hochschule Hannover, Hannover, Germany

**Keywords:** Bornavirus, variegated squirrel bornavirus 1 (VSBV-1), zoonotic infection, time-resolved phylogeny, surveillance, animal trade, seroprevalence study, squirrel

## Abstract

The variegated squirrel bornavirus 1 (VSBV-1) is a recently discovered emerging viral pathogen which causes severe and eventually fatal encephalitis in humans after contact to exotic squirrels in private holdings and zoological gardens. Understanding the VSBV-1 epidemiology is crucial to develop, implement, and maintain surveillance strategies for the detection and control of animal and human infections. Based on a newly detected human encephalitis case in a zoological garden, epidemiological squirrel trade investigations and molecular phylogeny analyses of VSBV-1 with temporal and spatial resolution were conducted. Phylogenetic analyses indicated a recent emergence of VSBV-1 in European squirrel holdings and several animal–animal and animal–human spill-over infections. Virus phylogeny linked to squirrel trade analysis showed the introduction of a common ancestor of the known current VSBV-1 isolates into captive exotic squirrels in Germany, most likely by Prevost’s squirrels (*Callosciurus prevostii*). The links of the animal trade between private breeders and zoos, the likely introduction pathway of VSBV-1 into Germany, and the role of a primary animal distributor were elucidated. In addition, a seroprevalence study was performed among zoo animal caretakers from VSBV-1 affected zoos. No seropositive healthy zoo animal caretakers were found, underlining a probable high-case fatality rate of human VSBV-1 infections. This study illustrates the network and health consequences of uncontrolled wild pet trading as well as the benefits of molecular epidemiology for elucidation and future prevention of infection chains by zoonotic viruses. To respond to emerging zoonotic diseases rapidly, improved regulation and control strategies are urgently needed.

## Introduction

Emerging viral pathogens, many of which emerge in clusters or outbreak scenarios and sometimes even as pandemics, are major threats to public and animal health. Owing to advances in next-generation sequencing (NGS) technology, the discovery of novel pathogens in human clinical cases or reservoir animal species has become more feasible. The variegated squirrel bornavirus 1 (VSBV-1; family *Bornaviridae*, species *Mammalian 2 orthobornavirus*) is such a recently discovered emerging viral pathogen, initially detected by NGS-based metagenomics. VSBV-1 was retrospectively detected in 2015 as the cause of fatal encephalitis in three male private breeders of exotic variegated squirrels (*Sciurus variegatoides*) in the East of Germany (cases in holdings A, B, and C). The three breeders had died in 2011–2013 [1]. *S. variegatoides* is a species of the Sciurinae subfamily of squirrels originating from Central America. In 2018, VSBV-1 was retrospectively found to be responsible for the fatal limbic encephalitis of a female zoo animal caretaker in the North of Germany who had died in 2013 (case D.2). She had previously cared for Southeast Asian Prevost’s squirrels (*Callosciurus prevostii,* subfamily Callosciurinae) in zoo D [2]. Epidemiological investigations in private holdings and zoological gardens in Germany and elsewhere in Europe revealed VSBV-1 detection rates of 1.5% in Sciurinae and 8.5% in Callosciurinae squirrels [3,4].

While the clinical disease [1,2], case definition [5] and immunopathology [6] have been described for human VSBV-1 encephalitis, the origin of VSBV-1 and its introduction into and spread within Germany, remain to be elucidated. A previous epidemiological study among private squirrel breeders in Germany demonstrated a complex squirrel trading network, with early animal imports from Costa Rica by an unknown German breeder [5]. However, the interconnection of the squirrel trade between the private sector and zoological gardens remained unclear.

Here we provide molecular and epidemiological evidence for Prevost’s squirrels as an early infected species during VSBV-1 spread in Germany, with a later transmission to captive Central American variegated squirrels in private holdings. Our study was initiated by the detection of a further human VSBV-1 encephalitis case in a male animal caretaker from zoo D. Epidemiological and serological investigations were performed in various German zoos in connection with this newly detected human case, showing complicated intermingling squirrel trading pathways between private breeders and zoological gardens. Furthermore, this study provides further insights into the spread of this novel viral pathogen among different exotic squirrel species in captivity and spill-over infections to humans.

## Materials and methods

### Genome recovery of VSBV-1 from newly detected human and exotic squirrel cases

The formalin-fixed paraffin-embedded (FFPE) brain biopsy of a 41-year-old male zoo animal caretaker (case D.1) with encephalitis was retrieved from a pathology archive, after the detection of bornavirus-reactive antibodies in the patient’s serum by an indirect immunofluorescence antibody test (IFAT) using a persistently infected cell line and a line blot assay with recombinant bornavirus phosphoprotein (P) antigen [1,2] in early 2019. The FFPE sample was tested in a VSBV-1-specific reverse transcription quantitative PCR (RT-qPCR) assay [1,2] and underwent unbiased NGS using a NextSeq550 Illumina sequencing platform for complete virus genome reconstruction as described elsewhere [7].

The patient had developed encephalitic symptoms in 2007 and underwent a brain biopsy in the same year. He had been suffering from chronic encephalitis/encephalopathy of unknown origin ever since. He had worked from 1996 to 2007 in the same zoo in Northern Germany (zoo D) where a female animal caretaker (case D.2) had died of VSBV-1 encephalitis in 2013 [2].

For complete VSBV-1 genome reconstruction from three VSBV-1 RT-qPCR-positive Prevost’s squirrels kept in zoos J and L in the Southwest of Germany, Sanger dideoxy-chain termination sequencing or NGS using the Ion S5 XL System with a 530 chip (Thermo Fischer Scientific, Waltham, MA, USA) was performed from squirrel brain tissue [8,9]. All other squirrel-derived VSBV-1 sequences included in this study were obtained previously [3,4] by the same methods. See Table 1 for an overview of the materials used in this study.

**Table 1.** Materials used in this study.

Human-derived VSBV-1 sequences^a^					
Nomenclature in the text	Employment (zoo or holding)	Year	Accession number	Tissue type/procedure	Reference
Case A	Holding A	2014	LN713681	brain autopsy	1
Case D.1	Zoo D	2007	MN092363	brain biopsy	this report
Case D.2	Zoo D	2013	MF5977762	brain autopsy	2
					
Human VSBV-1 serology					
Number of individuals	Employment (zoo or holding)	Year		Serological testing	Reference
26	Zoo D	2020		negative	this report
5	Zoo J	2020		negative	this report
6	Zoo *P*	2020		negative	this report
15	Zoo O	2020		negative	this report
1	Holding X	2020		negative	this report
Animal-derived VSBV-1 sequences					
Nomenclature in the text	Origin (zoo or holding)	Year	Accession number	Species/tissue type	Reference
#20_011	Zoo J	2019	MW234349	*Callosciurus prevostii* /CNS	this report
DH1_08-16	Zoo L	2016	KY508798	*Callosciurus prevostii* /CNS	this report
DH2_06-16	Zoo J	2016	KY508799	*Callosciurus prevostii* /CNS	this report

^a^ All three human VSBV-1 encephalitis cases were also seropositive.

Abbreviations: CNS: central nervous system; VSBV-1: variegated squirrel bornavirus 1.

### Analysis of phylogeny and spread of VSBV-1

In order to investigate the phylogenetic relationships and the spread of VSBV-1 in captive exotic squirrels in German holdings, two approaches were chosen:

First, a Bayesian maximum clade credibility (MCC) tree was reconstructed for the four newly determined VSBV-1 sequences together with all 22 complete coding VSBV-1 sequences with known time (year) and geographical origin available in GenBank using a Bayesian Markov Chain Monte Carlo (MCMC) approach available in BEAST v1.8 [10,11]. The temporal signal of the data set was visualized using regression of the root-to-tip divergence inferred from the maximum likelihood (ML) tree against the sampling time in TempEst [12]. For the MCC tree, we employed a relaxed uncorrelated log normal (UCLN) molecular clock, a flexible demographic model (coalescent Gaussian Markov Random field Bayesian Skyride model, GMRF) as the best demographic scenario detected, and the TN93+Γ model of nucleotide substitution. An asymmetric model with Bayesian Stochastic Search Variable selection (BSSVS) was applied to identify statistically significant transition rates between host species and geographic locations. An asymmetric model with a total of four MCMC chain lengths was run for 10^8^ generations (with 10% burn-in) with subsampling every 10^4^ iterations to achieve convergence as assessed by Tracer v1.5 [13]. The MCC trees were visualized using FigTree v1.4.1 (http://tree.bio.ed.ac.uk/software/figtree/). Given that the time of sampling of all VSBV-1 sequences included in this study was available, the temporal dynamics and the time to the most recent common ancestor (tMRCA) were estimated.

Second, a phylogenetic tree of the VSBV-1 nucleotide sequences was inferred using the ML method implemented in PhyML [14] with 1000 pseudo-replicates. The Akaike information criterion was chosen as the model selection framework, and the General Time-Reversible model of sequence evolution with gamma-distributed rate variation among sites (GTR + Γ) was chosen as the best model.

### Epidemiological investigations of the squirrel trade in zoos and private holdings

Focusing on zoo D in Northern Germany, where human cases D.1 and D.2 had been found infected in 2007 and 2013, respectively, all exotic squirrel trade movements were analysed in cooperation with the respective zoo veterinarians to determine the source of the VSBV-1 infection. Where possible, oral swabs, faecal or serum samples were obtained from the squirrels in these holdings and tested for VSBV-1 RNA or bornavirus-reactive antibodies as described [3,4].

Within both the private and the zoo sector, information was gathered about the history and the structure of exotic squirrel holdings in Germany and other European countries. Private holders of exotic squirrels were informed in media for small mammal enthusiasts and asked to provide the numbers and species of squirrels kept in their holdings. Concurrently, possible holders and breeders of squirrels in Germany were identified by internet searches on social media or selling platforms and private websites. If contact attempts were successful, holders were interviewed about the origin and whereabouts of their squirrels. Diagnostic molecular testing of squirrels and serological testing for owners were offered. Likewise, European zoos were informed about VSBV-1 via different zoo organizations (European Association of Zoo and Aquaria [EAZA], Deutsche Tierpark-Gesellschaft, Verband der Zootierärzte). Simultaneously, zoos keeping squirrels were identified by consulting zoo inventory databases (Zoological Information Management System and zootierliste.de) to contact them directly for interviews, and free testing of squirrels and staff was offered.

If available in the private owners’ records, those of the zoos, or in the European monitoring studbook for Prevost's squirrels compiled by the EAZA, the datasets for each variegated squirrel, Prevost's squirrel and other susceptible species on birth, entry, transfer, and exit dates were aligned to a temporal network and combined with testing results.

### Serological investigations for VSBV-1 infection of zoo animal caretakers and a private squirrel breeder

All present and past animal caretakers of zoo D, who had contact with exotic squirrels, and animal caretakers from other zoos that had exotic squirrel trading connections with zoo D (zoo O in Central Germany, zoo P in the West and zoo J in the Southwest of Germany), as well as a participating breeder, were serologically screened for bornavirus-reactive IgG antibodies by IFAT and the line blot assay.

The IFAT uses Crandell-Rees Feline Kidney (CRFK) cells persistently infected with BoDV-1 strain V and uninfected cells of the same cell line as controls. Due to high antigenic cross-reactivity within the genus Orthobornavirus, the IFAT also detects antibodies against VSBV-1 [1,2]. All sera with intranuclear IFAT patterns indicative for bornavirus infections [2] in dilutions ≥1:10 were regarded as positive. As confirmatory assay, a line blot with recombinant P protein from VSBV-1 was employed [2]. The cut-off of the line blot is 16 arbitrary units, as validated by the manufacturer (Euroimmun, Lübeck, Germany). In our study, sera from laboratory-confirmed human VSBV-1 encephalitis cases were used as positive controls, whereas a pooled serum of 20 healthy blood donors was used as negative control. The exact diagnostic sensitivity and specificity of the assays are so far unclear, as only few VSBV-1 encephalitis patients are known to date. However, all molecularly confirmed human VSBV-1 encephalitis cases from whom serum and/or cerebrospinal fluid was available, showed positive results in both the IFAT and line blot.

Present, but not past animal caretakers of zoo D had already been serologically tested for VSBV-1 infection in 2018 [2].

In addition, all participating animal caretakers and the breeder were interviewed for possible encephalitic episodes of themselves or fellow co-workers if known. Informed consent was obtained from all participants.

### Monitoring of squirrels within zoo D for VSBV-1 infection

The current group of remaining exotic squirrels in zoo D, Swinhoei’s striped squirrels (*Tamiops swinhoei*; subfamily Callosciurinae), was sampled on a regular basis. Oral swabs or faecal samples were collected and tested in February 2016, April 2018, July–August 2019 and November 2020. Complete carcasses of deceased Swinhoei’s striped squirrels were submitted for VSBV-1 RT-qPCR analysis in July 2017, April 2019 and November 2020.

## Results

### Genome recovery of VSBV-1 from newly detected human and exotic squirrel cases

VSBV-1-specific RT-qPCR from human case D.1’s archived FFPE brain sample was positive with a cycle threshold value of 30.9. Using NGS, a complete coding sequence was reconstructed (MN092363).

Complete VSBV-1 coding sequences were also generated from brain tissue of two Prevost’s squirrels kept in zoo J (2016 and 2019; GenBank accession numbers KY508799 and MW234349) and one Prevost’s squirrel in zoo L (2016; KY508798).

### Phylogeny of VSBV-1 and spread among exotic squirrels

**Figure 1.**
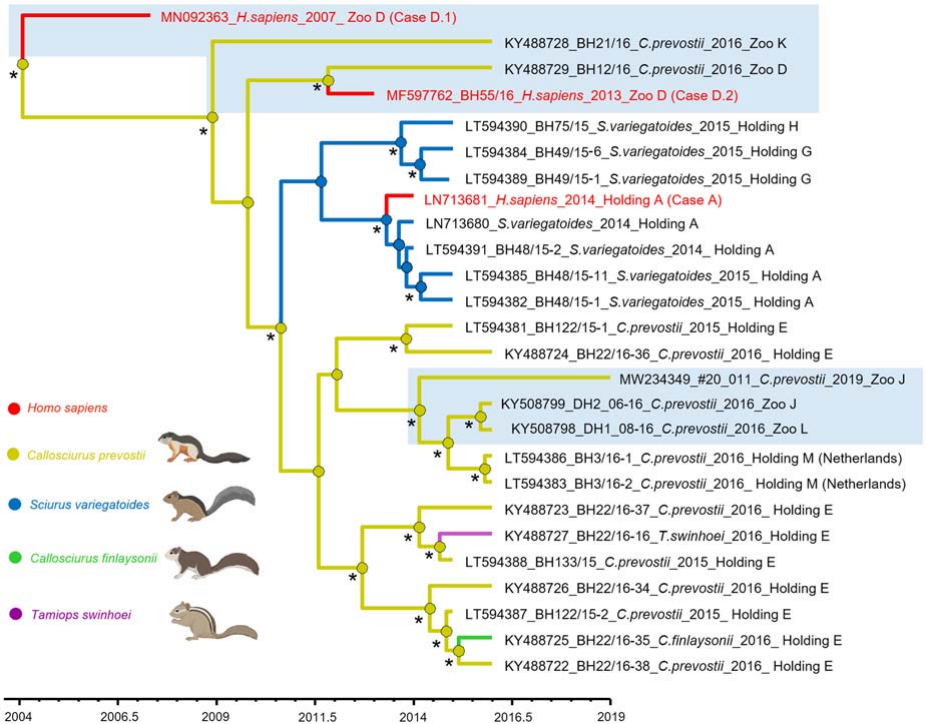
Bayesian maximum clade credibility (MCC) tree of VSBV-1 based on complete coding sequences showing the spread of the virus between different private holdings and zoos. Branches are coloured by the host and represent the transitions between different host species for the virus and the host of the common ancestor of all VSBV-1 strains, and the circle at the nodes the most probable host species of their descendant nodes (see colour codes). Spill-over infections to humans are shown in red. Human cases B and C are not included, as no complete virus genomes were available. Virus infections in zoos are shown in shaded areas. Statistical support of grouping from Bayesian posterior probabilities (clade credibility ≥90%) is indicated with an asterisk. Taxon information includes GenBank accession number, sample designation, host, year of detection and location of origin. Time in years is reported in the axis below the tree.

**Figure 2.**
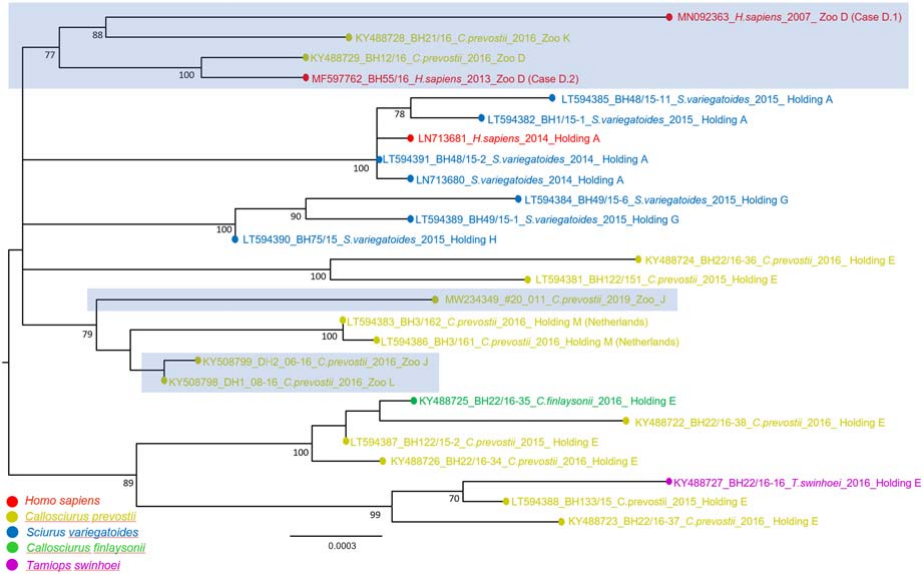
Maximum-likelihood (ML) phylogenetic tree of VSBV-1 based on complete coding sequences showing the phylogenetic placement of the human-derived strains compared with squirrel-derived strains. Sequence information is coloured to illustrate different hosts. Spill-over infections to humans are shown in red. Human cases B and C are not included, as no complete virus genomes were available. Virus infections in zoos are shown in shaded areas. ML bootstrap replicates scores (>70%) are shown next to the nodes. Taxon information includes GenBank accession number, sample designation, host, year of detection and location. Scale bar indicates nucleotide substitutions per site.

The phylogenetic trees inferred by both Bayesian MCC (Figure 1) and ML approach (Figure 2) of the complete coding VSBV-1 sequences showed their clustering in distinct groups defined by place of sampling (e.g. private holdings and zoos). Similarly, the human-derived VSBV-1 sequences clustered with the respective squirrel-derived virus sequences in specific local settings (holding A and zoo D), reflecting individual spill-over infections from squirrels to a private animal breeder (case A) and to two zoo animal caretakers (cases D.1 and D.2; Figure 1). The MCC approach suggested a single virus introduction in the captive exotic squirrel population, which led to subsequent virus transmissions in various private holdings and zoos. There were no hints for multiple introduction events of VSBV-1 into Germany. After the introduction, local transmissions among the animals ensued. Infections of variegated squirrels were linked to each other and were the result of a single transmission event from a Prevost’s squirrel in an unknown holding. Further transmissions, such as from Prevost’s squirrels to a Finlayson's squirrel as well as from Prevost’s squirrels to a Swinhoei's striped squirrel, occurred in holding E (Figure 1).

These Prevost’s squirrel-to-human, variegated squirrel-to-human, Prevost’s squirrel-to-variegated squirrel, Prevost’s squirrel-to-Finlayson's squirrel and Prevost’s squirrel-to-Swinhoei's striped squirrel transitions were supported by a posterior probability (pp.) of 95%. The most likely host species of the common ancestor of all VSBV-1 strains in Germany was the Prevost’s squirrel (pp. 92%).

The tMRCA was probably around 2003, with the 95% highest posterior density (HPD) interval for 1998–2007 (Figure 1).

### Epidemiological investigations of the squirrel trade in zoos and private holdings

**Figure 3.**
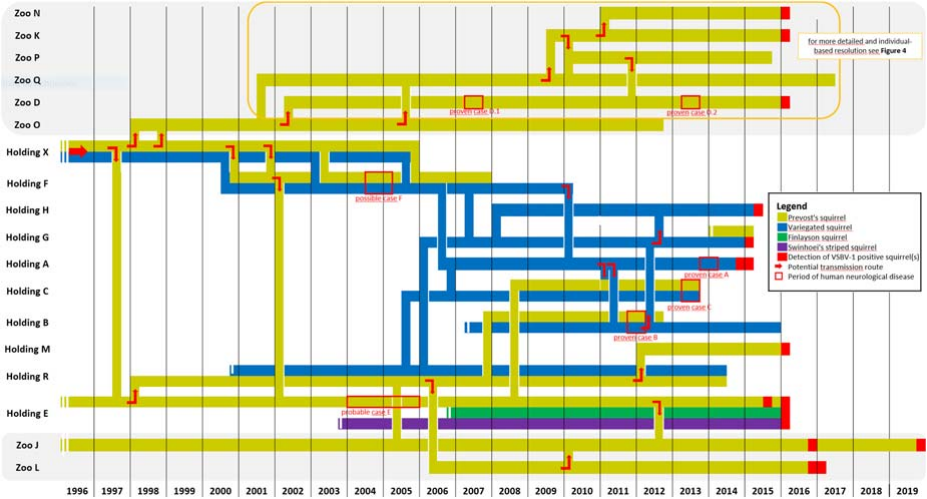
Spatio-temporal reconstruction of trade between zoos and private holdings with confirmed VSBV-1 positive squirrels. Timeline of squirrel presence and trading routes, including human cases and detection of VSBV-1-positive squirrels. Each horizontal line, differently coloured to illustrate the host species, represents the presence of at least one squirrel of that species at the named location over time. The vertical coloured lines indicate the trading route of one or more squirrels from one location to another at the distinct point in time. Squirrel holdings in zoos are shown in shaded areas. Filled red boxes on the coloured lines represent the quarter of the year in which one or more VSBV-1 positive squirrels were detected in the respective institution. Red frames mark the time period of reported neurological disease of proven, probable and possible human VSBV-1 encephalitis cases. Red arrows mark the assumed transfer of one or more infected squirrels. Streamlining was performed by omitting unaffected squirrels and holdings with no ties to affected animals and institutions, respectively. Affected holdings without reliable data were also not included.

**Figure 4.**
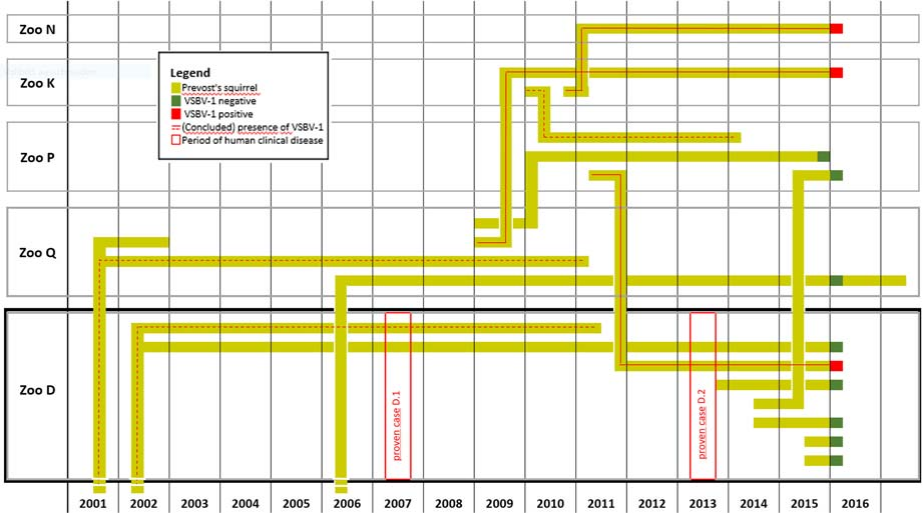
Presence of individual Prevost’s squirrels in zoo D and contact zoos aligned with the occurrence of the two occupation-related human cases. Each light green line represents the presence (horizontal) or the transfer (vertical) of one individual Prevost’s squirrel in or between involved zoos, which are depicted by black (zoo D) and grey frames. Filled red or dark green ends of the pathways mark positive and negative testing results for VSBV-1 RNA, respectively. Red frames indicate the period of symptom onset in human cases D.1 and D.2. Continuous red lines mark squirrels diagnosed with a VSBV-1-infection, dotted red lines represent squirrels assumed to be infected.

Based on information of squirrel owners and contact persons within small mammal specialist groups of private and zoo organizations, the formerly unknown German breeder [5], who had imported exotic squirrels to Germany (holding X) was located by repeated internet searches. This breeder X was interviewed on the history of his holding and the squirrel trading pathways. His contact dealers and holders were traced back, if possible, to identify animal origins and destinations as well as further contact animals and humans. Due to the lack of documentation for the majority of the identified private holders, exact trading data on individual squirrels were not available. Therefore, all individuals of one species in the same location were regarded as a single epidemiological unit, and dates of movements were reconstructed as far as possible at least to the level of a quarter of the year. Combined with the records of the zoo institutions and the studbook, a squirrel trading network was drafted (Figures 3 and 4).

The interviews with the owner of holding X, a 55-year-old male German, revealed that he had started keeping and breeding exotic squirrels in the late 1970s in Western Germany at the age of 12 years. Thereafter, he bought his first Prevost’s squirrels from Indonesia (Sumatra and Borneo) imported by a Dutch animal broker, from whom he obtained additional Prevost’s squirrels in the following decades, lastly in 1997. In 1990, variegated squirrels imported from Nicaragua by another animal distributor were included in his husbandry, which consisted then of about 150–200 animals of more than ten species. He bred and sold the animals and their offspring during the whole period mainly to German and Dutch private holders as well as to few German and Swiss zoos. In 1998, he emigrated to Costa Rica, where he ran a squirrel breeding farm with variegated squirrels and Prevost’s squirrels both introduced from his former German holding. Throughout the years in Germany and Costa Rica, he collaborated closely with the owner of holding F, a 65-year-old male German squirrel breeder, who died of encephalitis of unknown cause in 2005 (classified as possible VSBV-1 encephalitis case [5]). To him, he had left the majority of his squirrels before his emigration. The import of squirrels from Costa Rica and their further distribution was mainly conducted by breeder F. In holding F, the squirrels were reportedly kept under crowded conditions and transport boxes were used multiple times for different species without cleaning. Holding F repeatedly sold variegated squirrels to the owners of holding A (who died of proven VSBV-1 encephalitis in 2011 [1]) and holding G, from where they or their offspring were further distributed (Figure 3).

In addition, the former holding X repeatedly sold Prevost’s squirrels to zoo O. In 2002, two animals were transferred from zoo O to zoo D, where patient D.1 had been working since 1996 and cared for the zoo’s Prevost’s squirrel holding until the onset of his VSBV-1 encephalitis in 2007. One of the two founder animals tested negative for VSBV-1 by RT-qPCR of brain tissue and for bornavirus-reactive antibodies in 2015, the other one had died in 2011 and could not be tested. This latter squirrel is assumed to be the source of the patient’s VSBV-1 infection since no other squirrels had been present in the holding between 2002 and 2007 (Figure 4). In 2011, zoo D had introduced additional Prevost´s squirrels from zoo P, which had received the founder male of its population from zoo K. One of the squirrels transferred from zoo P to zoo D tested positive for VSBV-1 in 2016 (Figure 4; KY488729 in Figures 1 and 2) and is assumed to be the source of infection of human case D.2 (MF597762 in Figures 1 and 2).

Holding X frequently traded Prevost’s squirrels to the owner of holding E since 1993, when holder E bought his first breeding couple from breeder X. Holder E, who kept the largest number of VSBV-1 positive animals found in a single holding, had a transient neurological episode in 2004 and is the only known living seropositive individual to date (classified as a probable VSBV-1 encephalitis case [5]). After breeder X’s emigration, the trade continued via holding F. The last Prevost’s squirrels were moved to holding E via this route in 2002. Holding E in turn traded the animals and their offspring to a growing number of further holdings, including zoo J in 2012, and a holding with several breeding couples (holding R) in 1998. Two Prevost’s squirrels found positive in 2016 in a Dutch private holding (holding M) and one Prevost’s squirrel found positive in zoo L in 2017 originated from holding R. According to former contacts, various species of exotic squirrels were kept at holding R. Unfortunately, the owner of holding R was not willing to participate in the investigations. In 2019, he stated that he had abolished the holding in the aftermath of the first detections of VSBV-1 in Prevost’s squirrels in Germany in 2017.

Based on trading information, the likeliest time period of introduction of VSBV-1 into Germany via holding X is in or before 1997. The slow spread of the virus into various other holdings is attributed to transfers of single infected animals, followed by spill-over infections to other captive exotic squirrel species.

### Serological investigations for antibodies to VSBV-1 in zoo animal caretakers and a private squirrel breeder

A total of 52 present and past animal caretakers of zoo D (*n* = 26), zoo J (*n* = 5), zoo P (*n* = 6) and zoo O (*n* = 15), who had had contact with exotic squirrels, participated in the study. Testing for bornavirus-reactive antibodies was negative for all participants. With the exception of the already known cases D.1 and D.2, no encephalitic episodes of the study participants or of fellow coworkers were reported.

The serum of owner of holding X, who had been repeatedly bitten and scratched by Prevost’s and variegated squirrels on his farm, was negative for bornavirus-reactive antibodies. He never developed any neurological episodes.

### Monitoring of squirrels within zoo D for VSBV-1 infection

A group of four Swinhoei’s striped squirrels was kept at the same time as the Prevost’s squirrels in zoo D in a neighbouring building. All oral swabs and faecal samples collected from the whole group between February 2016 and November 2020 tested negative for VSBV-1 RNA. Brain samples from three animals of this group that had died in 2017, 2019, and 2020 were likewise negative.

## Discussion

In this study, we aimed to elucidate the possible origin of VSBV-1 in captive squirrel holdings in Europe and its transmission within and between multiple squirrel species in these holdings and from there to humans. Infected squirrels are asymptomatic and show high viral loads not only in the central nervous system but also in organs capable of secretion and excretion (kidneys, urinary bladder, skin, and oral cavity), thus qualifying them as potential natural reservoirs [1-4]. However, the origin of the virus and its route of introduction into the captive squirrel populations in Europe remained to be elucidated.

The striking genetic homogeneity of all currently known VSBV-1 genomes suggests a single introduction of the virus into the known captive exotic squirrel populations in Europe, which is supported by consolidation of phylogenetic analysis and trade network investigations in this study. The Bayesian analysis estimates the most recent common ancestor of the VSBV-1 strains in Europe to have occurred in 1998–2007 (probably around 2003), which is in line with the recorded segregation of the different arms of the trade network in 1997 from holding X. Based on these observations, the introduction of VSBV-1 into German holdings is likely to have occurred prior to 1997 in holding X. Phylogenetic data indicate that the initially infected animals in the here identified network were Prevost´s squirrels, suggesting a possible Southeast Asian origin of VSBV-1 and an introduction via the import of this species.

Examinations of wild squirrel populations and investigations of human encephalitis cases of unknown aetiology for VSBV-1 infection in Southeast Asia are currently under way and may shed more light on the epidemiology and origin of VSBV-1. However, we cannot exclude that Prevost's squirrels do not represent the original host, but have become infected by transmission from another species, either in holding X, in an unidentified holding in Europe or elsewhere prior to its import. Furthermore, the virus may have originated from a single introduction from an unknown local squirrel or non-squirrel reservoir in Europe. However, recent studies failed to detect VSBV-1 in 77 wild Eurasian red squirrels (*Sciurus vulgaris*) from Germany and the UK [3,4]. VSBV-1 was not found in free-ranging invasive populations of Pallas’ squirrels (*Callosciurus erythraeus; n* = 35) from Italy and Eastern grey squirrels (*Sciurus carolinensis*; *n* = 83) from the UK [15]. Moreover, in this study, we did not detect the virus in further squirrels in zoo D.

We here provided a broader view on the complex trading connections and animal interchanges of Prevost's squirrels and variegated squirrels, the two most relevant species for VSBV-1 spread in Europe. Among other breeders, we were able to locate and interview the primary distributor of squirrel species in Germany. Furthermore, the trading links between private holdings and zoological gardens in Germany, which could not be elucidated in a previous study [5], were now uncovered. The results show that zoos had kept predominantly Prevost’s squirrels, whereas private breeders partly had kept variegated squirrels and Prevost’s squirrels in close proximity, resulting in an enhanced probability of cross-species transmission. This transmission might have occurred in private holding F, from which suboptimal animal housing conditions were reported. From there, the virus was likely introduced into several other private variegated squirrel holdings (Figures 3 and 4).

In 2004, the first probable human case (holder E) occurred, followed by a possible human case in 2005 (holder F), and the first proven case (D.1) in 2007. Both human occupation-related fatal encephalitis cases (D.1 and D.2) had independently occurred in the same rather small zoological garden in Northern Germany six years apart. However, the two different animals suspected as the respective sources of infection had originated from two different zoos. The Prevost’s squirrel suspected to be responsible for the spill-over infection to case D.1 in 2007 originated directly from zoo O. It had died untested in 2011, but was identified as the only possible source of infection of case D.1. The squirrel assumed to be responsible for the transmission event to patient D.2 in 2013 originated from zoo P, but its (possibly VSBV-1-infected) ancestors could be traced back via zoo K also to zoo O (Figure 3). This is also reflected by phylogenetic analyses, which show that the human and squirrel sequences from zoo D form a monophyletic clade with the squirrel sequence from zoo K (Figure 2). All six Prevost’s squirrels from this zoo were euthanized and underwent a detailed pathological examination [3]. In the five squirrels found to be negative, neither in the brain nor in any other tissue genetic evidence for an infection with VSBV-1 was found by RT-qPCR. As orthobornaviruses are known to cause persistent infections and there is no hint in the literature on the possibility of clearance of the virus, it is highly unlikely that one of these animals was infected earlier. The additional analysis of samples from other squirrel species kept in the same facility as the VSBV-1-infected Prevost´s squirrels revealed no positive results. Extensive serological studies in Prevost’s squirrels were not performed. Such studies are hampered by the circumstance that the procedure of obtaining serum is connected with a high risk of sudden death of the animals. In the few cases where sera were available, the results of RT-qPCR of buccal swabs or brain tissue and serology were congruent.

Interestingly, the sequences of the strains originating from the two different squirrels from zoo J sampled in 2016 and 2019 also suggest two independent virus introductions from German holdings. Again, this is corroborated by the trade analysis, as zoo J received animals from at least three sources with confirmed or suspected VSBV-1 infections (zoo L, holdings E and R). The relative heterogeneity of the seven strains from Prevost’s squirrels originating from holding E (Figure 1) may have been due to the relatively long circulation of the virus in this holding after its likely introduction in 1997 until 2016.

Holder X and all zoo animal caretakers from affected zoos, who participated in the study tested negative for bornavirus-reactive antibodies. Although the presence of antibodies below the detection limit cannot be completely ruled out, there was no anamnestic evidence of a past neurological disease. The only so far detected living seropositive person is breeder E, who reported to have recovered after having suffered from a chronic neurological disease in 2004/05 [5]. The finding of few seropositive humans suggests a high-case fatality rate of VSBV-1 infections, as noted also for the clinically similar human Borna disease virus 1 infections [16].

In conclusion, exotic pet trade is an important driver of the spread of exotic pathogens to new environments promoting contact with susceptible new hosts. This study demonstrates the spread of VSBV-1 as an emerging pathogen in captive squirrels and spill-over infections to humans. Understanding the epidemiology is crucial to develop and implement surveillance strategies for control. Our report highlights the risk of VSBV-1 transmission in private and occupational settings and the need for monitoring of all exotic squirrel holdings. Annual testing of oral swabs and faecal samples by RT-qPCR is currently recommended [5] in order to identify and remove any infected animals. Furthermore, this study underlines the general necessity for an official pet trading database, which may provide a valuable tool to contact risk groups rapidly and efficiently in the case of a zoonotic outbreak.
